# Management of breakthrough disease in patients with multiple sclerosis: when an increasing of Interferon beta dose should be effective?

**DOI:** 10.1186/1471-2377-11-26

**Published:** 2011-02-25

**Authors:** Luca Prosperini, Giovanna Borriello, Laura De Giglio, Laura Leonardi, Valeria Barletta, Carlo Pozzilli

**Affiliations:** 1 Multiple Sclerosis Centre, Dept. of Neurology and Psychiatry, S. Andrea Hospital, Sapienza University, Rome, Italy

## Abstract

**Background:**

In daily clinical setting, some patients affected by relapsing-remitting Multiple Sclerosis (RRMS) are switched from the low-dose to the high-dose Interferon beta (IFNB) in order to achieve a better control of the disease.

**Purpose:**

In this observational, post-marketing study we reported the 2-year clinical outcomes of patients switched to the high-dose IFNB; we also evaluated whether different criteria adopted to switch patients had an influence on the clinical outcomes.

**Methods:**

Patients affected by RRMS and switched from the low-dose to the high-dose IFNB due to the occurrence of relapses, or contrast-enhancing lesions (CELs) as detected by yearly scheduled MRI scans, were followed for two years. Expanded Disability Status Scale (EDSS) scores, as well as clinical relapses, were evaluated during the follow-up period.

**Results:**

We identified 121 patients switched to the high-dose IFNB. One hundred patients increased the IFNB dose because of the occurrence of one or more relapses, and 21 because of the presence of one or more CELs, even in absence of clinical relapses. At the end of the 2-year follow-up, 72 (59.5%) patients had a relapse, and 51 (42.1%) reached a sustained progression on EDSS score. Overall, 85 (70.3%) patients showed some clinical disease activity (i.e. relapses or disability progression) after the switch.

Relapse risk after increasing the IFNB dose was greater in patients who switched because of relapses than those switched only for MRI activity (HR: 5.55, p = 0.001). A high EDSS score (HR: 1.77, p < 0.001) and the combination of clinical and MRI activity at switch raised the risk of sustained disability progression after increasing the IFNB dose (HR: 2.14, p = 0.01).

**Conclusion:**

In the majority of MS patients, switching from the low-dose to the high-dose IFNB did not reduce the risk of further relapses or increased disability in the 2-year follow period.

Although we observed that patients who switched only on the basis on MRI activity (even in absence of clinical attacks) had a lower risk of further relapses, larger studies are warranted before to recommend a switch algorithm based on MRI findings.

## Background

Three formulation of Interferon beta (IFNB) are nowadays approved as disease modifying agents for subjects with relapsing-remitting (RR) Multiple Sclerosis (MS): IFNB-1b 250 mcg subcutaneous every other day (s.c., e.o.d.) (Betaferon, Bayer Schering, Berlin, Germany); IFNB-1a 30 mcg intramuscular once weekly (i.m., o.w.) (Avonex, Biogen Idec, Cambridge, USA); IFNB-1a 22 or 44 mcg subcutaneous three times per week (s.c., t.p.w.) (Rebif, Merck Serono, Geneva, Switzerland). Large randomised trials demonstrated that IFNB reduces the frequency of MS attacks and the number of new lesions on magnetic resonance imaging (MRI) [1-6]. Also, post-marketing studies suggest a long-term benefit of IFNB treatment on disease activity and disability progression [7-10]. Nevertheless, a considerable number of patients experience a breakthrough disease, i.e. evidence of clinical or imaging disease activity or progression despite the IFNB treatment [11]. Therefore, a significant proportion of subjects requires treatment switching or escalation in order to avoid accumulation of irreversible disability.

To date, there is no evidence to guide an alternative therapy in patients with breakthrough disease [11]. However, in daily clinical setting some patients starting with the low-dose (i.m., o.w. 30 mcg, or s.c., t.p.w. 22 mcg IFNB-1a) are routinely switched to the high-dose IFNB (s.c., e.o.d. 250 mcg IFNB-1b, or s.c., t.p.w. 44 mcg IFNB-1a) in case of breakthrough disease, according to suggestions coming from therapeutic recommendation of consensus groups [12,13].

Two head-to-head studies comparing i.m. 30 mcg IFNB-1a o.w. with s.c. 250 mcg IFNB-1b e.o.d. and 44 mcg IFNB-1a t.p.w. (INCOMIN and EVIDENCE, respectively) provided evidences that naive patients started with the high-dose IFNB had a better outcome than those receiving a low dose regimen [14,15]. It has also been suggested that increasing the IFNB dose may be useful in reducing mean relapse rate and the accumulation of subclinical lesions as detected on MRI [16,17]. However, these studies have not investigated whether the criteria adopted to switch patients to the high-dose IFNB could have an influence on the clinical outcomes.

The 2-year clinical outcomes of patients switched from the low-dose to the high and/or more frequently administered dose of IFNB are reported in the present observational study. Moreover, we attempted to identify which patients with breakthrough disease could benefit from an increase of the IFNB dose.

## Methods

### Study design and participants

Patients affected by RRMS [18] according to Poser [19] or McDonald Criteria [20], and switched to a more frequently administered and/or higher dose of IFNB (Betaferon or Rebif 44) in consequence of breakthrough disease during the low-dose IFNB regimen (Avonex or Rebif 22) were included in this 2-year, independent, observational, post-marketing study at the MS Centre of S. Andrea Hospital in Rome. There was no well-defined protocol for switching patients to the high-dose IFNB (occurrence of one or more relapses and/or MRI activity), but it was a decision taken by the neurologist depending on the growing availability over years of new drugs active against MS (i.e., Natalizumab, experimental treatment, etc).

Patients were regularly followed-up from the start of the IFNB treatment; clinical and MRI data were prospectively collected and stored into an electronic database (after obtaining an informed consent by each patient). The local Ethical committee board provides exemption of approval for post-marketing prospective studies.

**Figure 1:**
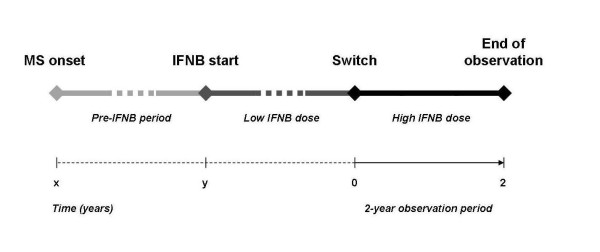
**Study design with relative time-points**. Note that pre-IFNB and low IFNB dose periods had a different duration for each patient, while after switch all patients were followed for 2 years.

The study design with relative time-points are described in Figure 1. We defined as "switch" the time-point at which patients interrupted the low-dose and started the higher IFNB dose; therefore, we defined a pre-switch period (i.e., the time frame elapsed between the start of the IFNB treatment and the switch) and a 2-year observational post-switch period.

Patients receiving a low-dose IFNB only for titration, or those discontinuing the IFNB treatment mainly for poor tolerance, or those with previous exposure to immunomodulant or immunosuppressive agents were excluded from this study.

Clinical data, including the occurrence of relapses and the Expanded Disability Status Scale (EDSS) score [21], were evaluated for each subject every 3 months. Unscheduled visits were also performed in suspect of relapse or any other clinically relevant condition.

An exacerbation was defined as the appearance or reappearance of one or more symptoms attributable to MS, accompanied by objective deterioration on neurological examination lasting at least 24 hours, in the absence of fever and preceded by neurological stability for at least 30 days [19,20].

We also collected yearly brain and spinal cord MRI since the start of treatment with IFNB, focusing on the absence/presence of MRI activity (i.e. the detection of at least one contrast-enhancing lesions); additional MRI scans were performed, if necessary. Clinical examination (including EDSS score) and MRI scan were done in stable patients (i.e. at least 30 days after the last assumption of steroids administered for relapses).

### Outcome measures

We considered two main outcomes over the 2-year observation period: (a) the occurrence of at least one relapse, and (b) a sustained progression of 1 or more points on EDSS score (starting after the switch and confirmed in two consecutive neurological visits separated by at least a 6-month interval).

### Statistical analysis

All values are expressed as a mean ± standard deviation (± SD) or median (range), as appropriate. Differences between groups were tested by using the Chi-square test and the U Mann-Whitney test.

Two Cox proportional hazards models were built for identifying the predictors of experiencing a relapse or a sustained progression on EDSS score after the switch. As main time variable for time-to-event analyses we considered the interval (in years) elapsed between the switch and last visit, or high IFNB dose discontinuation, or outcome reach, whichever came first.

Gender, age, MS duration, EDSS score, presence/absence of an active MRI (each considered at the time of switch), pre-IFNB annualised relapse rate, IFNB type (Betaferon or Rebif 44), as well as duration of initial IFNB treatment, number of relapses, and EDSS change occurred during the low-dose IFNB regimen were included as covariates in each model. Variables were added in the models in a forward stepwise fashion, and interactions terms were tested, where appropriate. All models were stratified by IFNB type received by patients before the switch (Avonex or Rebif 22).

Data have been analysed by using the Statistical Package for Social Sciences, version 16.0 (SPSS, Chicago, IL, USA).

## Results

### Participants

We examined 283 patients starting the low-dose IFNB formulation from October 1997 to March 2008 (Avonex, n = 120; Rebif 22, n = 163). Mean (SD) duration of IFNB treatment was 4.3 (2.3) years, and mean annualised relapse rate was 0.48 (range 0-3). A total of 108 (38.1%) patients had a sustained EDSS increase during IFNB treatment.

Out of these 283 patients, 121 patients switched to the high-dose IFNB, while 162 continued to receive the low-dose IFNB. Table 1 shows the demographic and clinical characteristics of patients who switched to the high-dose IFNB and those who did not. At the start of treatment with IFNB there were no differences between the two groups.

**Table 1:** Demographic and clinical characteristics of 283 patients starting a low IFNB dose.

	Patients switching to high IFNB dose n = 121	Patients remaining on low IFNB dose n = 162	Pooled n = 283
Male gender, n (%)	39 (32.2)	49 (30.2)	88 (31.1)

Age, years	31.5 (8.5)	32.8 (9.8)	32.2 (9.2)

MS duration, years	5.4 (5.3)	6.1 (5.9)	5.8 (5.7)

Annualised relapse rate pre-IFNB	1.00 (0.74)	0.92 (0.76)	0.95 (0.75)

EDSS score, median [range]	1.5 [0 - 3.5]	1.5 [0 - 3.5]	1.5 [0 - 3.5]

Active MRI scan, n (%)	65 (53.7)	69 (42.6)	134 (47.3)

All value are expressed as mean (standard deviation), unless indicated otherwise.

All p-values are > 0.05

Among the 121 patients who increased the IFNB dose, therapy switching included: Rebif 22→Rebif 44 (n = 59, 47.9%), Avonex→Rebif 44 (n = 46, 38.4%), Avonex→Betaferon (n = 16, 13.7%). After the switch to the high-dose IFNB, 72 (59.5%) patients experienced a relapse, and 51 (42.1%) patients reached a sustained progression on EDSS score. Overall, 85 (70.3%) patients showed some clinical disease activity (i.e. relapse or disability progression) over the 2-year observation period after the switch.

### Risk of relapses or worsening in disability after the switch

One hundred (82.6%) patients switched to the high-dose IFNB due to MS attacks (40 also had an active MRI scan at switch), while 21 (17.4%) patients switched due to evidence of activity on MRI scan, but not relapses, during the low-dose IFNB regimen.

Only 5 patients (23.8%) who were switched on the basis of MRI activity experienced a further relapse, while 67 (67.0%) of patients switching because of relapses had a further exacerbation over the 2-year after the increase of IFNB dose (p < 0.001).

No significant differences in proportion of patients reaching a sustained progression on EDSS score were observed between patients who increased the IFNB dose because of relapses and those who switched only on the basis of MRI (p = 0.08).

According to the Cox model, when compared with patients switched only on the basis of MRI activity (i.e. no relapses during the low-dose IFNB regimen), those relapsing were more likely of having a further relapse even after the switch (HR: 5.55, 95% C.I. 2.22 - 13.85, p = 0.001). Moreover, this risk of relapsing during the 2-year observational period was related with a younger age at switch (HR: 0.94, 95% C.I. 0.91 - 0.97; p < 0.001), and the number of clinical bouts occurred before the switch: one bout confers a risk ratio of 3.56 (95% C.I. 1.34 - 8.42; p = 0.01), two or more bouts a risk ratio of 7.89 (95% C.I. 3.10 - 19.85; p < 0.001) (see Table 2). The risk of further relapses was not increased in patients who switched to the high-dose IFNB because of a combination of clinical and MRI activity at switch.

**Table 2:** Final model for the stepwise Cox hazard model analysis showing Hazard Ratio (with relative confidence intervals and p-values) for relapsing after the increase of IFNB dose.

	HR	95% C.I.	p-value
Male gender	0.68	0.39 - 1.16	0.1

Age at switch (each year)	0.94	0.91 - 0.97	<***0.001***

No. of relapses occurred during the low-dose IFNB regimen			
0 (*)	Ref.	-	-
1	3.56	1.34 - 8.42	***0.01***
≥ 2	7.89	3.10 - 19.85	<***0.001***

IFNB type (Betaferon or Rebif 44)	1.14	0.52 - 2.51	0.7

IFNB: Interferon Beta; EDSS: Expanded Disability Status Scale

(*) this value refers to patients switched only on the basis of an active MRI scan

The variables predictive for reaching a sustained disability progression after the switch were the EDSS score at baseline (HR: 1.77, 95% C.I. 1.39 - 2.26; p < 0.001), and the combination of clinical and radiological activity at switch (HR: 2.14, 95% C.I. 1.16 - 3.96; p = 0.01) (see Table 3).

**Table 3:** Final model for the stepwise Cox hazard model analysis showing Hazard Ratio (with relative confidence intervals and p-values) for worsening in disability after the increase of IFNB dose.

	HR	95% C.I.	p-value
Male gender	1.68	0.88 - 2.13	0.1

Age at switch (each year)	0.98	0.94 - 1-02	0.3

*Interaction term *(relapse * active MRI scan at switch)	2.14	1.16 - 3.96	***0.01***

EDSS score at switch (each point)	1.77	1.39 - 2.26	***< 0.001***

IFNB type (Betaferon or Rebif 44)	0.83	0.28 - 2.85	0.7

IFNB: Interferon Beta; EDSS: Expanded Disability Status Scale.

## Discussion

Results from the present study suggest that the risk of having a relapse despite the increase of IFNB dose raised according to the number of clinical attacks occurred during the assumption of the low-dose IFNB, and it is reduced when the switching was based on MRI findings rather than on clinical activity. Moreover, the chance of being progression-free after the switch is related to a lower EDSS score and the absence of clinical and MRI activities. At this regard, we may assume that in some patients an incomplete recovery from relapse occurred, with a substantial influence on their EDSS scores.

Overall, less than 1/3 of our patients remained free from clinical disease activity (i.e. absence of relapse and sustained progression on EDSS score) over the 2-year observational period following the increase of IFNB dose. This could imply that the majority of switchers should be considered poor responder to IFNB therapy regardless the dose and/or frequency of administration, and further supports the hypothesis that a treatment strategy encompassing the increase of IFNB dose should be useful only in some selected cases.

After the increase of IFNB dose, the majority of patients switched only for the evidence of MRI activity had a better clinical outcome than those switched because of the occurrence of relapses. Therefore, we might suggest that monitoring the effect of IFNB treatment with regular MRI scans is recommendable even in absence of clinical relapses.

It has been known that conventional MRI represents a powerful tool to monitor latent disease activity, providing a measurable and sensible marker of response to IFNB therapy [22-25]. However, we cannot exclude that patients switched only on the basis of MRI findings might also have had good outcomes without switching to the high-dose IFNB, as the absence of a control group. At this regard, Rio and colleagues showed that patients with only MRI activity and no relapses in the first year of IFNB treatment did not experience an increase of relapses or disability over a 3-year follow-up [26].

Although randomized clinical trials demonstrated a more pronounced effect of high-dose, high-frequency IFNB when compared with both the low-dose, equal-frequency [1,27] and the low-dose, low-frequency regimens [14,15] in naïve patients, data on the effectiveness of increasing the IFNB dose in patients with breakthrough disease are scarce. The open-label extension phase of the EVIDENCE study, involving 223 patients converted from Avonex to Rebif 44, documented a 50% reduction in the annualised relapse rate after the switch [16]. However, we must consider also that in the EVIDENCE study all patients originally randomized to Avonex were offered to receive Rebif 44, independently from the response status during the blind phase of the study. While the authors suggested that increasing the IFNB-1a dose and frequency could rapidly reduce ongoing disease activity, they cannot discharge the hypothesis that the significant reduction in relapse rate might be due, at least in part, to the regression to the mean phenomenon.

Some open-label studies exploring the usefulness of switching among immunomodulating drugs had different designs and provided conflicting results [28-30]. Two studies reported a decrease in both relapse rate and proportion of relapse-free subjects after the switch [29,30], whilst the QUASIMS study did not provide any support of more favourable outcomes after switching from an IFNB formulation to another [28]. One possible explanation of this discrepancy is that these observational surveys considered different subtype of switch (i.e. from IFNB-1a to IFNB-1b and *viceversa*, from IFNB to GA, etc.); also, these studies were not specifically aimed to determine the crude effect of an increase of the IFNB dose. Furthermore, in some studies the patients were switched on the basis of tolerability rather than a persistent disease activity.

In the present study, patients had variable periods of observation before and after the switch, thus precluding any attempt to estimate the effectiveness of an increase of the IFNB dose in suppressing disease activity and slowing disability progression.

Being an observational report, our study suffers from other limits, such as the small sample size, the unavailability of control group, blindness and randomization, as well as the lack of data on neutralising antibodies (NAbs) against IFNB. However, there is evidence that NAbs presence could explain only the 20% of the suboptimal response to IFNB treatment [31].

Despite these limits, our study might contribute to define a therapeutic algorithm to manage breakthrough disease in patients on treatment with a low-dose IFNB. The identification of patients with subclinical disease activity during the low-dose IFNB treatment, and an early switch to the high-dose IFNB, seem to be effective in achieving a better control of the disease. On the contrary, when relapses occurred during the pre-switch period, especially in combination with MRI activity, patients did not seem benefit from the increase of the IFNB dose in the following years.

Since efficacy of GA has been demonstrated comparable to IFNB [32-34], switching among immunomodulating treatments may represent an interesting approach in case of treatment failure [35,36]. The scientific rationale for switching to other therapies is strongest for patients on IFNB therapy with persistent high-titre of NAbs [37]. However, a more aggressive approach (Natalizumab or Mitoxantrone) is warranted for patients at high risk of accumulation of fixed disability or with shorter intervals between attacks [13].

## Conclusions

We suggest neurologists to consider the presence of sub-clinical activity, as detected on MRI, in the decision to switch a patient from the low-dose to the high-dose IFNB, even in absence of clinical relapses. Further efforts are warranted to clearly define whether MRI findings might be considered a key element in the choice of increase the IFNB dose when the response to the low-dose IFNB is suboptimal.

## List of abbreviations

RRMS: relapsing-remitting multiple sclerosis; EDSS: Expanded Disability Status Scale; MRI: magnetic resonance imaging; CELs: contrast enhancing lesions; IFNB: Interferon beta.

## Competing interests

The authors declare that they have no competing interests.

## Authors' contributions

LP and CP had full access to the data, and take the final responsibility for the content of the present manuscript. LP and CP are responsible for concept and design of the study, contributed to data analysis and manuscript drafting; GB and LL contributed to data analysis and interpretation and manuscript revision; LDG, LL and VB collected the data. All authors read and approved the final manuscript.

## Pre-publication history

The pre-publication history for this paper can be accessed here:

http://www.biomedcentral.com/1471-2377/11/26/prepub
